# Carbon: Nitrogen Interaction Regulates Expression of Genes Involved in N-Uptake and Assimilation in *Brassica juncea* L.

**DOI:** 10.1371/journal.pone.0163061

**Published:** 2016-09-16

**Authors:** Parul Goel, Monika Bhuria, Mamta Kaushal, Anil Kumar Singh

**Affiliations:** 1 CSIR-Institute of Himalayan Bioresource Technology, Palampur-176061 (HP), India; 2 Academy of Scientific and Innovative Research, New Delhi, India; National Taiwan University, TAIWAN

## Abstract

In plants, several cellular and metabolic pathways interact with each other to regulate processes that are vital for their growth and development. Carbon (C) and Nitrogen (N) are two main nutrients for plants and coordination of C and N pathways is an important factor for maintaining plant growth and development. In the present work, influence of nitrogen and sucrose (C source) on growth parameters and expression of genes involved in nitrogen transport and assimilatory pathways was studied in *B*. *juncea* seedlings. For this, *B*. *juncea* seedlings were treated with four combinations of C and N source *viz*., N source alone (-Suc+N), C source alone (+Suc-N), with N and C source (+Suc+N) or without N and C source (-Suc-N). Cotyledon size and shoot length were found to be increased in seedlings, when nitrogen alone was present in the medium. Distinct expression pattern of genes in both, root and shoot tissues was observed in response to exogenously supplied N and C. The presence or depletion of nitrogen alone in the medium leads to severe up- or down-regulation of key genes involved in N-uptake and transport (*BjNRT1*.*1*, *BjNRT1*.*8*) in root tissue and genes involved in nitrate reduction (*BjNR1* and *BjNR2*) in shoot tissue. Moreover, expression of several genes, like *BjAMT1*.*2*, *BjAMT2* and *BjPK* in root and two genes *BjAMT2* and *BjGS1*.*1* in shoot were found to be regulated only when C source was present in the medium. Majority of genes were found to respond in root and shoot tissues, when both C and N source were present in the medium, thus reflecting their importance as a signal in regulating expression of genes involved in N-uptake and assimilation. The present work provides insight into the regulation of genes of N-uptake and assimilatory pathway in *B*. *juncea* by interaction of both carbon and nitrogen.

## Introduction

Nitrogen (N) is one of the essential macronutrients for plants. The requirement of N in plants is higher as compared to other nutrients as it is important constituent of amino acids, RNA, DNA and chlorophyll [1]. The process of N-assimilation is tightly linked with carbon (C) assimilation [2]. The interaction between these two pathways is important for plant growth and development [3–5]. Plants have developed complex sensing and signalling mechanisms in order to adapt to change in environmental factors including nutrient availability. The C and N treatments can exert both, antagonistic and synergistic effects on plant at morphological (lateral root growth) and physiological (endogenous sugar, chlorophyll content) levels [6–7]. It has been also observed that C and N balance affects the plant phenotype [7]. The coordination between these two nutrients regulates various developmental and metabolic processes in plants.

**Fig 1:**
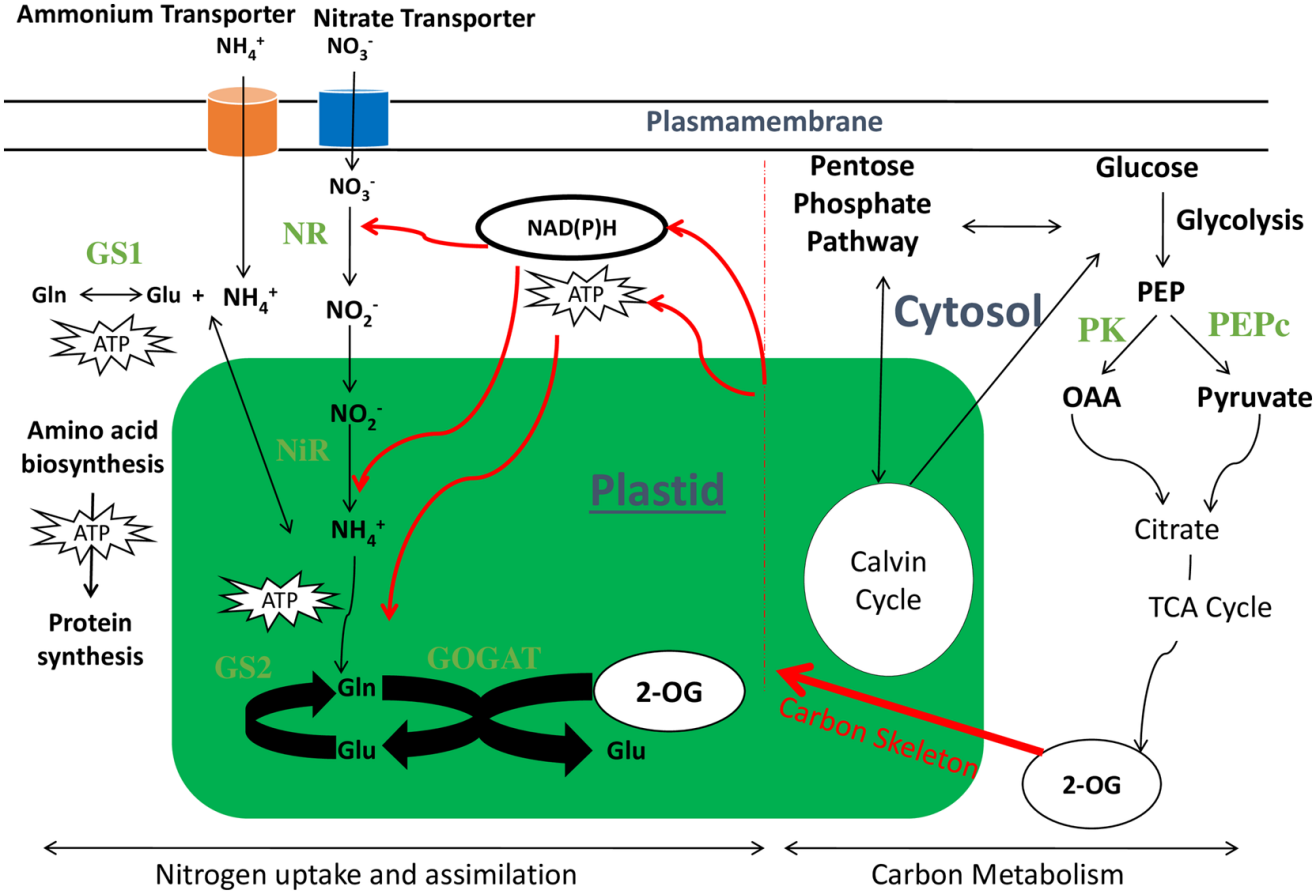
Interaction of Carbon and Nitrogen pathways. The carbon metabolic pathway provides energy (ATP) and reducing potential NAD(P)H to nitrogen assimilation process. Moreover, the carbon skeleton part in amino acids also comes from 2-OG (2-oxoglutarate) of TCA (Tricarboxylic acid cycle) cycle. Nitrate transporter (NRT), Ammonia transporter (AMT), Nitrate Reductase (NR), Nitrite Reductase (NiR), Glutamine Synthetase isoforms (GS1, GS2), Glutamate Synthase (GOGAT), Glutamate (Glu), Glutamine (Gln), 2-OG (2-Oxoglutarate), Phosphoenol pyruvate (PEP), Pyruvate kinase (PK), Oxaloacetic acid (OAA).

In plants, the uptake of N (either nitrate or in ammonium form) takes place by specialized transporters present in roots [8]. The reduction of nitrate can take place in cytoplasm of both, root and shoot tissues. Once the nitrate is reduced into nitrite, its further reduction into ammonium takes place in chloroplast. The final incorporation of ammonium ions into amino acids also takes place in chloroplast. The process of carbon assimilation mainly occurs in leaves [9]. Thus, leaves are the site where both, N and C assimilation processes interact. The assimilated products of both C and N are then distributed to various plant parts to complete growth cycle [10]. The process of N assimilation, especially when nitrate is its main source is energetically costly process and requires large amount of ATP and C-skeleton produced from the carbon metabolic processes [11]. On the other hand, large amount of N is essential for plant photosynthetic machinery and also in CO_2_ assimilation. This reflects that proper coordination and interaction of both these pathways is critical for the plant growth and development (Fig 1).

The C and N both can act as potent signalling molecules to regulate expression of several genes involved in N and C metabolism and photosynthesis etc. [12,4]. As a signal, nitrate not only regulates the expression of nitrate related genes [13], but also regulates expression of carbon metabolizing genes [14]. Addition of nitrate in N depleted plants leads to the direct induction of genes of glycolytic pathway (phosphoglycerate mutase and Glc-6-phosphate isomerase), pentose phosphate pathway, like glucose-6-phosphate dehydrogenase (G6PDH), 6-phosphogluconate dehydrogenase (6PGDH) and genes of organic acid pathway, phosphoenolpyruvate carboxylase (PEPC), pyruvate kinase (PK) that provide C-skeleton to nitrate assimilation process for amino acid production [13–16]. Genes encoding for nitrate transporter, nitrate reductase and glutamine synthetase were found to be induced in sugar depleted *Arabidopsis*, when supplied with exogenous sugar [17,18]. Sugar also induces the expression of NRT genes in *Arabidopsis* [19,20]. Moreover, reduction in sugar level leads to inhibition of nitrate assimilation in tobacco plants expressing antisense rubisco (RBCS) construct [21].

The C and N pathways interact and coordinate with each other in order to regulate the gene expression. Expression of several genes was found to be influenced by status of C and/or N nutrients [22,23]. Several microarray studies have been performed to identify the genes and pathways regulated by interaction between C and N signalling. Effect of glucose and inorganic nitrogen on *Arabidopsis* seedlings was studied using microarray, which revealed that glucose regulates several genes including those involved in nitrogen assimilation, nitrogen metabolism and carbohydrate metabolism as compared to inorganic nitrogen [18]. Genome-wide microarray analysis of *Arabidopsis* seedlings in response to different C and N treatments has identified the involvement of several putative *cis*-elements in the promoter region of the genes for mediating C/N responsive gene expression [24]. Qualitative network modelling has been performed to identify networks and sub-networks that respond to C, N or CN in *Arabidopsis* root system [25]. The three genes, nitrate transporter (*LIN1/NRT2*.*1*), glutamate receptor (*GLR1*.*1*) and a methytransferase named Oversensitive to sugar 1(*OSU1*) were found to be involved in CN signalling in *Arabidopsis* [26–28].

Economic importance of *B*. *juncea* as an oilseed crop is well known. Effect of elevated CO_2_ and moisture stress on C/N distribution was studied in *B*. *juncea* and showed that nitrogen content was reduced in plants under these growth conditions [29]. However, another study showed that addition of N is crucial for growth of *B*. *juncea* under elevated CO_2_ conditions [30]. Recently, the interactive effect of elevated CO_2_ and temperature on C/N metabolism was studied in *B*. *juncea* [31]. This study showed that activities of several C-metabolizing enzymes (phosphophenol-pyruvate carboxylase, malate dehydrogenase etc.) and N-metabolizing enzymes (glutamine synthetase, nitrate reductase and nitrite reductase etc.) were higher at elevated CO_2_ and combined treatment of elevated CO_2_ and temperature. However, no study has been performed to assess the effect of various C/N treatments on growth and transcriptional response of various C/N pathway genes in *B*. *juncea*. Therefore, the present work was performed to study the effect of C/N availability on seedling growth and also to identify genes of N and C pathways that are differentially regulated by either C/N independently or by interaction of both of these nutrients in root and shoot tissues, separately. We have studied various parameters, including root and shoot fresh weight and dry weight, root length, shoot length, chlorophyll and anthocyanin content under different availabilities of C and N source in the medium. Our results showed that C as a signal also plays an important role in regulating expression of genes of N-uptake and assimilatory pathway in both root and shoot tissues. In addition, we have identified several genes of N-uptake and assimilation pathway that are regulated by the interaction of C and N and thus may have an important role in C/N signalling pathway in *B*. *juncea*. Our study provides the molecular basis to the fact that coordination between C and N is important for plant growth and development.

## Materials and Methods

### Plant material and measurements of growth parameters

Healthy seeds of *B*. *juncea* cv. Varuna were surface sterilized with 70% ethanol for 2–3 minutes and then washed with autoclaved distilled water for 2–3 times. To study seedling growth and development, the seeds of *B*. *juncea* were germinated on modified MS medium (Murashige and Skoog) [32] with different availabilities of C (0% or 3% sucrose) and N source (0 mM or 1 mM KNO_3_/1 mM NH_4_NO_3_). In first condition, the medium was devoid of both C and N source (-Suc-N), in second condition, only C source and no N source was provided in medium or *vice versa* (+Suc-N or -Suc+N) and in third case, both carbon and nitrogen sources were present in the medium (+Suc+N). The seedlings were grown on appropriate medium in petriplates in culture room at 16h/8h photoperiod of photon flux density 115 mmolm^-2^s^-1^ at 22±2°C with 50% relative humidity. Fresh weight and length of root and shoot were measured after 10d of treatment. For dry weight measurement, the root and shoot tissues were oven dried for 48h at 80°C and cooled down to room temperature. The experiment was performed in triplicate with nine seedlings under each treatment.

### Chlorophyll content estimation

Chlorophyll was measured by the method given by Arnon [33]. The 100 mg of fresh plant material was extracted in 1 ml pre-chilled 80% acetone and centrifuged at 5000 rpm for 5 min. The supernatant was transferred to the fresh tube and remaining residues were homogenised again with pre-chilled 80% acetone, centrifuged and supernatant was transferred to the tube. The procedure was repeated until the chlorophyll was extracted completely. The final volume was made to 5 ml with 80% acetone. The absorbance was measured at 645 nm and 663 nm wavelength.

The chlorophyll a, b and total chlorophyll were calculated according to the Arnon equation:

$$Chl\;a\;\left(mg\;g^{-1}\right)\;=\;\left[\left(12.7\;\times \;A663\right)\;-\;\left(2.6\;\times \;A645\right)\right]*V/1000*W$$

$$\;Chl\;b\;\left(mg\;g^{-1}\right)\;=\;\left[\left(22.9\;\times \;A645\right)\;-\;\left(4.68\;\times \;A663\right)\right]*V/1000*W$$

$$Chl\;a+b\;\left(mg\;g^{-1}\right)\;=\;20.2\;\left(A645\right)\;+\;8.02\;\left(A663\right)\;*V/1000*W$$

Where, A: Absorbance at particular wavelength, V: Volume of extract, W: Fresh weight of plant tissue

### Anthocyanin content estimation

Total anthocyanin content was determined by method of Mancinelli et al. [34] with some modifications. The 100 mg of homogenized material was extracted in 300 μl of 7% (v/v) hydrochloric acid in methanol. In the extract, 200 μl of deionised water was added and mixed well. Chloroform (500 μl) was added to each sample to make a final volume of 1000μl and centrifuged at high speed for 2 min. The supernatant was transferred to a fresh tube and 600 μl of 1% (v/v) HCl in methanol was added and then centrifuged. The absorbance was measured at 530 nm and 657 nm.

### Gene expression analysis using qRT-PCR

To perform gene expression analysis, *B*. *juncea* seedlings were treated with various C/N concentrations as described by Oliveira and Coruzzi [23]. After emergence of true leaves, the seedlings were transferred to fresh MS media without any C and N source and acclimatized for 2d, thereafter seedlings were transferred to fresh MS media supplemented with or without sucrose and/or with or without N source as described above. After 8h of treatment, root and shoot tissues were harvested, frozen in liquid nitrogen and stored at -80°C for future use.

Total RNA from 100 mg tissue was extracted using iRIS method [35] and checked on formaldehyde denaturing gel. The 3μg of total RNA was used for first strand cDNA synthesise using Revert-Aid H Minus Reverse Transcriptase kit (Thermo Scientific, USA). In our previous study, we have cloned genes of NRT1, NRT2, AMT, NR, NiR, GS, GOGAT, GDH and ASN family from *B*. *juncea* and their sequence were submitted to Genbank [36]. The two genes involved in carbon metabolic pathway namely, phosphophenolpyruvate carboxylase (PEPC) and pyruvate kinase (PK) were also cloned and sequences were submitted to Genbank with accession no. KX352389 and KU214449, respectively. The primers for real-time PCR were designed using Primer Express software version 3.0.1. The details of gene specific primers used for the present study are shown in S1 Table. The qRT-PCR reactions were performed as described earlier [36]. In order to normalise the mRNA abundance, ubiquitin gene (*Ubq9*) was used as an internal control [37]. The qRT-PCR reactions were performed with three technical and three biological replicates. The relative expression value expressed as fold change was calculated according to 2(-delta delta CT) method [38]. The mean value of relative fold change was used to generate heat maps and to plot bar diagrams. Error bars in bar diagrams represent ± standard deviation. The genes with a fold change ≥2 or ≤0.5 (p-value <0.05) were considered as significantly up- or down-regulated.

## Results

### Effect of carbon and nitrogen on growth of *B*. *juncea* seedlings

**Fig 2:**
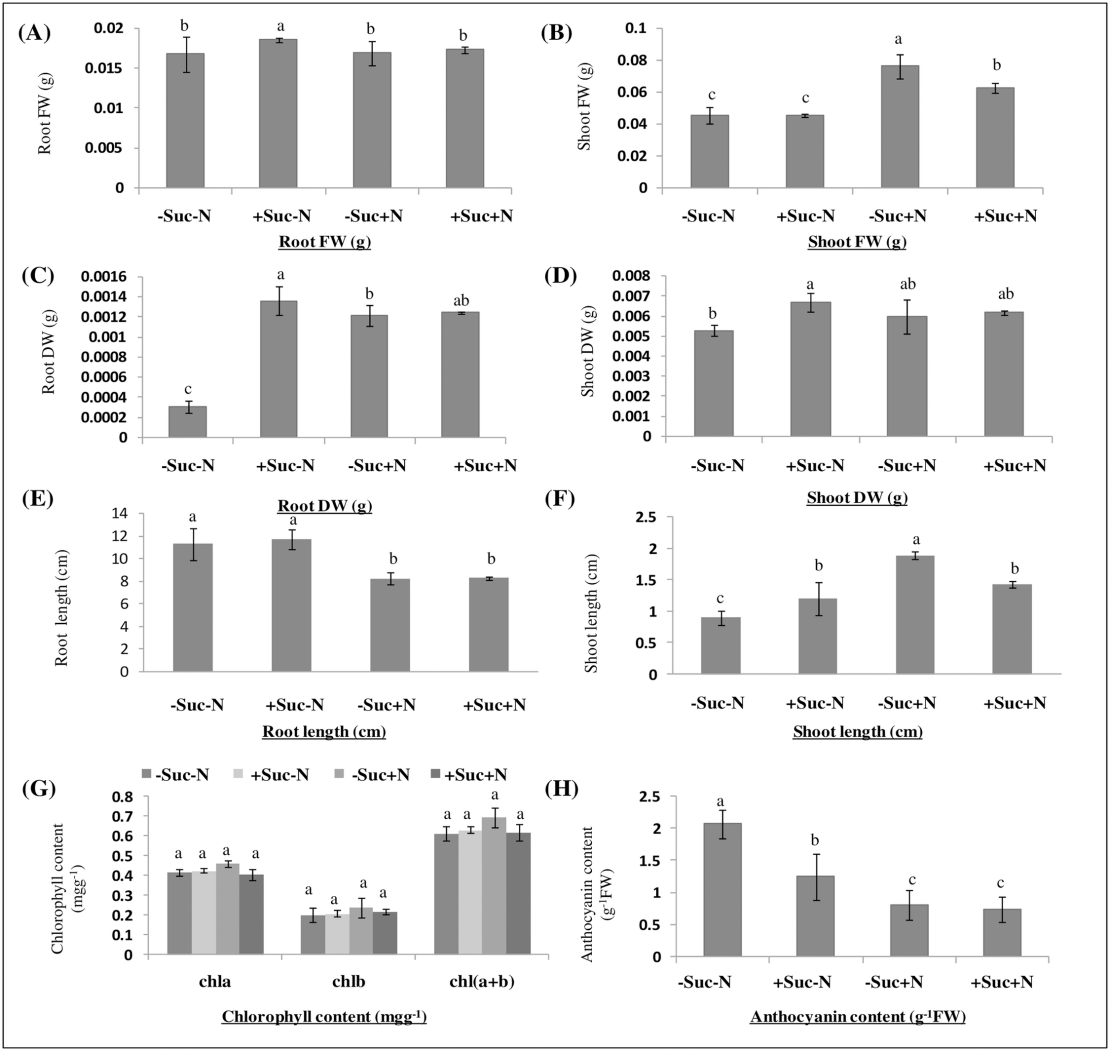
Effect of Carbon: Nitrogen availabilities on (A) root fresh weight (B) shoot fresh weight (C) root dry weight (D) shoot dry weight (E) root length (F) shoot length (G) chlorophyll content and (H) anthocyanin content in *B*. *juncea* seedlings. The vertical bars represent the mean ±SD of three independent experiments (n = 3) with nine seedlings under each treatment. Different letters on the top of the bars indicate significant difference at a level of P<0.05 by ANOVA using Duncan’s Multiple Range Test (DMRT).

To study the effect of carbon (C) and nitrogen (N) on *B*. *juncea*, the seedlings were grown on four different modified MS mediums with different combinations of C and N availability (-Suc-N, +Suc-N, -Suc+N and +Suc+N) (S1 Fig). It has been observed that seedlings grown on only N source (-Suc+N) had higher cotyledon size and growth as compared to seedlings grown on C source alone (+Suc-N) (S1 Fig). In case of root tissue, slightly higher fresh weight was observed in +Suc-N medium as compared to rest of the conditions (Fig 2A). However, in case of shoot tissue, maximum fresh weight was observed in -Suc+N medium followed by +Suc+N medium, while shoot fresh weight was found to be similar in -Suc-N and +Suc-N medium (Fig 2B). The maximum root and shoot dry weight was observed in +Suc-N medium followed by +Suc+N and -Suc+N medium (Fig 2C and 2D). The root and shoot lengths were also found to be affected under different conditions (Fig 2E and 2F). The mean primary root length was found to be decreased from an average of 11 cm on -Suc-N and +Suc-N medium to 8 cm on -Suc+N and +Suc+N medium (Fig 2E). However, increase in average shoot length from 0.9 cm on -Suc-N medium to an average of 1.9 cm was observed on -Suc+N medium (Fig 2F). However, no significant difference was observed in total chlorophyll content in seedlings under different carbon and nitrogen availabilities (Fig 2G). Seedlings grown on -Suc-N and +Suc-N medium exhibited purple coloration in leaves. The levels of anthocyanin in seedlings grown on -Suc-N media and +Suc-N media were found to be higher than in seedlings grown on +Suc+N and -Suc+ N medium (Fig 2H).

### Effect of nitrogen alone on expression of genes

**Fig 3:**
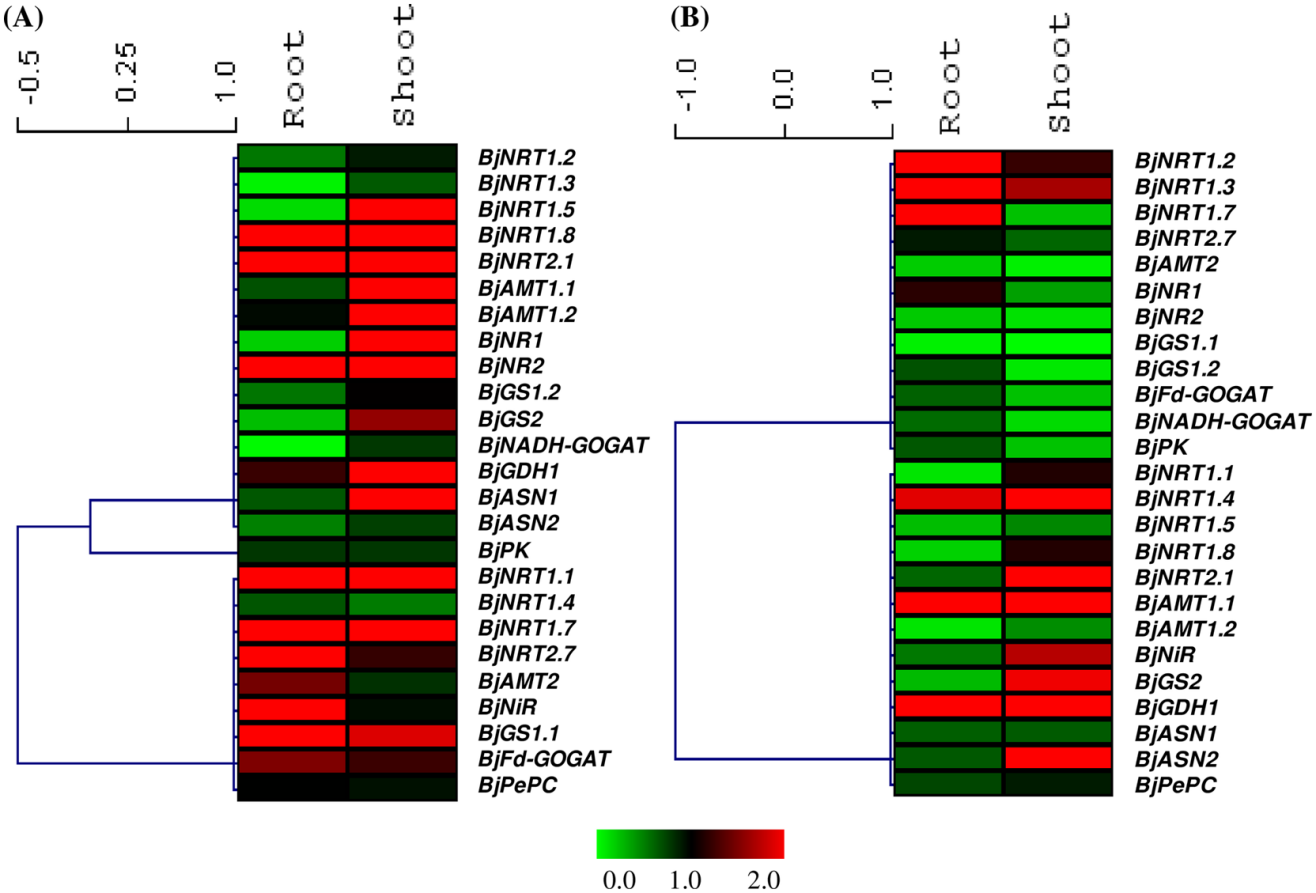
Heat maps showing relative expression of genes involved in nitrogen uptake, assimilation and gene of organic acid metabolism in root and shoot tissue of *B*. *juncea* in presence of N alone (-Suc+N) w.r.t -Suc-N condition (A) and in absence of N source alone (+Suc-N) w.r.t +Suc+ N condition (B). The vertical bars indicate relative expression ratio where red, black and green represent up-regulation, no change and down-regulation in transcript levels, respectively.

**Fig 4:**
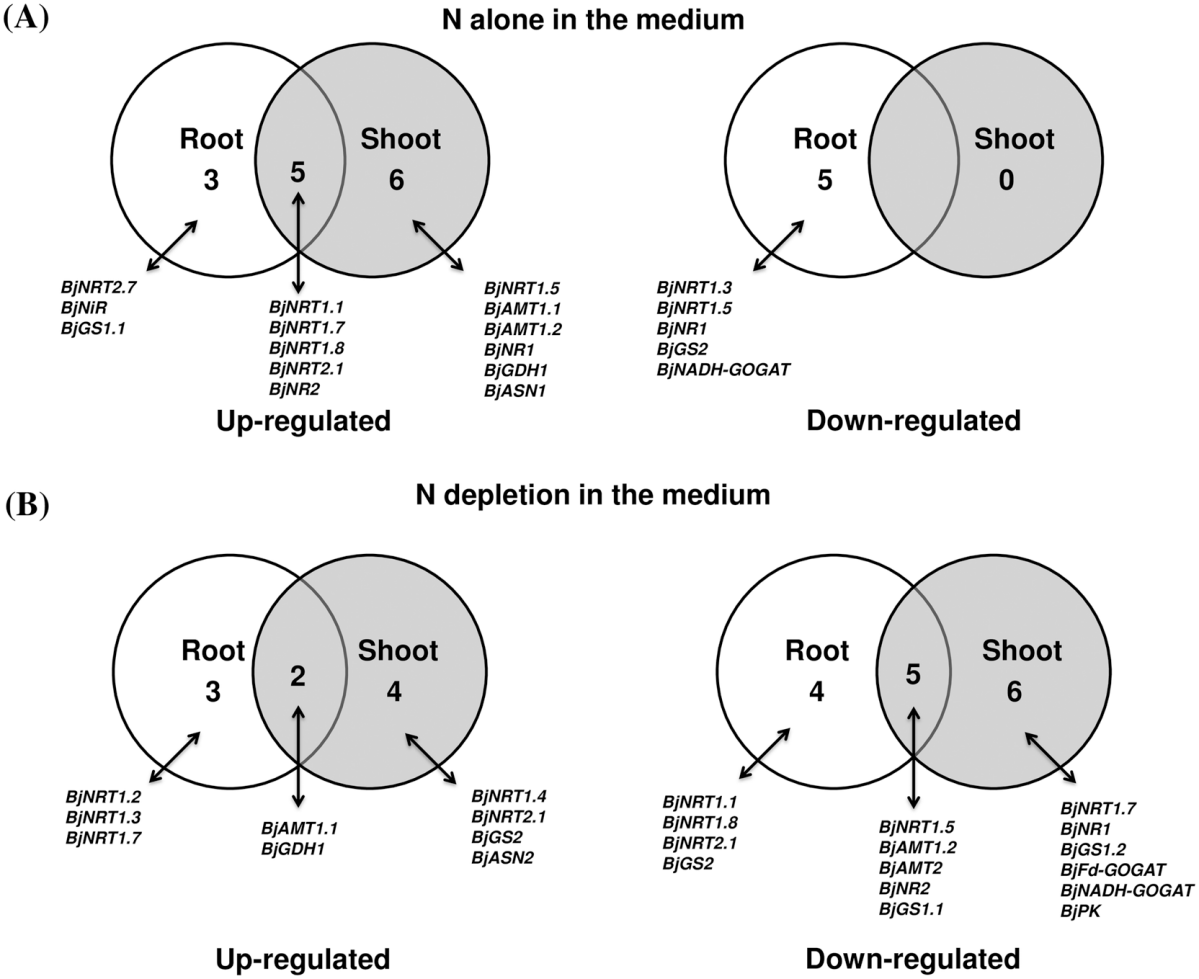
Venn diagram showing number of genes up-regulated or down-regulated in root and shoot tissues in presence of Nitrogen (N) alone in the medium (A), depletion of N in the medium (B). Genes with a relative fold change ≥2 and ≤0.5 with respect to control conditions were considered as up-regulated and down-regulated, respectively.

In order to study the effect of nitrogen source alone on the expression of genes involved in N-uptake and metabolism *viz*. genes encoding for low and high affinity nitrate transporters (NRT1 and NRT2), ammonium transporters (AMT), nitrate reductase (NR), nitrite reductase (NiR), glutamine synthetase (GS), glutamate synthase (GOGAT), glutamate dehydrogenase (GDH) and asparagine synthetase (ASN) and gene of organic acid metabolism *viz*. Pyruvate kinase (PK) and Phosphoenolpyruvate carboxylase (PEPC), we compared expression of genes in -Suc+N condition with -Suc-N condition. Root and shoot tissues showed differential expression pattern in response to exogenous application of nitrogen source (Fig 3A, S2A and S2B Fig, Table 1). The expression of thirteen genes in root (eight induced and five repressed) and eleven genes (all induced) in shoot tissue were significantly (P<0.05) modulated by nitrogen alone (Fig 4A, Table 1). The expression of majority of genes encoding nitrate transporters *viz*. *BjNRT1*.*1*, *BjNRT1*.*7*, *BjNRT1*.*8*, *BjNRT2*.*1*, and *BjNRT2*.*7* was upregulated in presence of N source alone in root tissue (Fig 3A, S2A Fig, Table 1). Whereas, the expression of *BjGS1*.*2*, *BjGS2*, *BjNADH-GOGAT* and *BjASN1* that are mainly involved in N assimilation was found to be either unaltered or downregulated in presence of N source in root tissue (Fig 3A, S2B Fig, Table 1). In case of shoot tissue, no significant downregulation in the expression of genes was observed. The expression of several genes, like *BjNRT1*.*1*, *BjNRT1*.*7*, *BjNRT2*.*1*, *BjAMT1*.*1*, *BjAMT1*.*2*, *BjNR1*, *BjNR2*, *BjGDH1* and *BjASN1* was found to be induced in presence of N source alone (Fig 3A, S2A and S2B Fig, Table 1). However, the expression of *BjPK* and *BjPEPC* gene was not significantly modulated in both root and shoot tissues in presence of nitrogen source alone.

**Table 1:** Relative expression values (Fold change with p-value) of 25 genes under different conditions as determined by qRT-PCR. The genes with relative fold change ≥2 and P-value <0.05 were considered as significantly upregulated and genes with relative fold change ≤0.5 and P-value <0.05 were considered as significantly downregulated.

Gene Name	Gene Description	Fold Change (P-value)							
		-Suc-N vs. -Suc+N		+Suc+N vs. +Suc-N		-Suc-N vs. +Suc-N		-Suc-N vs. +Suc+N	
		Root	Shoot	Root	Shoot	Root	Shoot	Root	Shoot
*BjNRT1*.*1*	Low affinity nitrate transporter 1.1	37.73 (0.0005500)	3.88 (0.0001634)	0.09 (2.45796E-07)	1.13 (0.1011076)	21.33 (2.51782e-05)	5.72 (0.0098899)	37.63 (0.0005500)	5.18 (0.0001634)
*BjNRT1*.*2*	Low affinity nitrate transporter 1.2	0.53 (0.1876643)	0.89 (0.0522700)	2.02 (0.0007311)	1.21 (0.2938386)	1.89 (0.1120836)	0.91 (0.0093977)	1.4 (0.1876643)	0.77 (0.0522700)
*BjNRT1*.*3*	Low affinity nitrate transporter 1.3	0.04 (2.1576E-05)	0.65 (0.0117653)	4.54 (0.0033815)	1.65 (0.0267036)	0.77 (0.2534806)	0.73 (0.3376850)	0.07 (2.15763E-08	0.46 (0.0117653)
*BjNRT1*.*4*	Low affinity nitrate transporter 1.4	0.66 (1.0923E-08)	0.51 (0.0012104)	1.89 (0.0028111)	3.32 (0.0007194)	0.78 (0.0226874)	0.715 (0.8639911)	0.41 (1.09232E-08)	0.2 (0.0012104)
*BjNRT1*.*5*	Low affinity nitrate transporter 1.5	0.13 (5.54354E-05)	5.23 (2.62069E-06)	0.25 (2.73006E-06)	0.46 (0.0004577)	0.92 (0.8353094)	0.35 (0.0008907)	4.35 (5.54354E-05)	0.63 (2.62069E-06)
*BjNRT1*.*7*	Low affinity nitrate transporter 1.7	4.41 (0.0106260)	3.13 (0.0047059)	2.28 (0.0045117)	0.24 (4.47306E-06)	18.83 (0.0001256)	1.88 (0.0177302)	2.16 (0.1062601)	6.53 (0.0047059)
*BjNRT1*.*8*	Low affinity nitrate transporter 1.8	7.19 (0.0085252)	10.66 (0.0033089)	0.17 (0.0045117)	1.13 (0.0172860)	8.83 (0.0002763)	1.16 (0.1410583)	3.43 (0.0085252)	1.12 (0.0033089)
*BjNRT2*.*1*	High affinity nitrate transporter 2.1	4.96 (0.0080825)	13.9 (0.0373262)	0.45 (2.867752E-06)	4.39 (0.0000879)	37.93 (0.0002763)	10.18 (0.0024960)	8.57 (0.0080825)	10.18 (0.3732623)
*BjNRT2*.*7*	High affinity nitrate transporter 2.7	10.37 (0.01942689)	1.2 (0.2471040)	0.89 (0.0000311)	0.59 (0.0006015)	5.23 (0.0000854)	0.78 (0.0022294)	1 (0.9426854)	1.34 (0.2471040)
*BjAMT1*.*1*	Ammonium transporter 1.1	0.68 (0.3274504)	3.16 (0.0197729)	2.33 (0.0396662)	3.05 (0.0013322)	0.81 (0.0192112)	1 (0.4149189)	1.52 (0.3274504)	0.16 (0.0197729)
*BjAMT1*.*2*	Ammonium transporter 1.2	0.96 (0.0003464)	3.37 (0.0420776)	0.09 (0.0007259)	0.44 (0.0048017)	5.19 (0.0081271)	0.67 (0.3478225)	7.9 (0.0003464)	6.04 (0.0720776)
*BjAMT2*	Ammonium transporter 2	1.45 (0.0013849)	0.81 (0.0003748)	0.19 (1.3149137)	0.05 (1.7701299E-08)	2.31 (0.0086450)	0.22 (0.1659745)	24.55 (0.0013849)	3.26 (0.0003748)
*BjNR1*	Nitrate reductase 1	0.18 (9.02597E-06)	3.55 (8.31672E-06)	1.16 (2.22546E-06)	0.37 (0.0001144)	6.08 (0.0134139)	1.18 (0.1659745)	5.19 (9.02597–06)	2.06 (8.31672E-06)
*BjNR2*	Nitrate reductase 2	2.14 (0.0012281)	6.07 (0.0008379)	0.2 (0.4748712)	0.11 (1.7035269E-07)	3.14 (0.00020780)	2.11 (1.98144E-05)	15.08 (0.0012281)	3.54 (0.0008379)
*BjNiR*	Nitrite reductase	12.43 (0.0008407)	0.94 (8.75703–05)	0.52 (0.0000403)	1.72 (6.0591156E-11)	2.34 (0.0002078)	0.53 (0.1139866)	5.06 (0.0008407)	0.54 (8.75703E-05)
*BjGS1*.*1*	Glutamine synthetase 1.1	2.25 (0.0001360)	1.86 (0.6860795)	0.05 (0.0078338)	0.01 (1.5164722E-12)	0.35 (0.0001243)	0 (0.0265427)	0.42 (0.0001360)	0.38 (0.0486079)
*BjGS1*.*2*	Glutamine synthetase 1.2	0.54 (0.0119553)	1.01 (0.0015690)	0.67 (5.98987E-09)	0.08 (1.7124235E-08)	1.13 (0.2372632)	1.38 (0.0010003)	1.95 (0.0119553)	0.55 (0.0015690)
*BjGS2*	Glutamine syntthetase 2	0.25 (0.0080836)	1.58 (0.0022519)	0.27 (0.0503371)	2.04 (0.0239502)	2.42 (0.0008845)	0.79 (0.0151489)	0.53 (0.0080836)	4.28 (0.0022519)
*BjFd-GOGAT*	Ferrodoxin dependent-glutamate synthase	1.5 (6.60402E-08)	1.23 (0.0055082)	0.61 (8.37534E-08)	0.24 (1.1399044E-06)	0.39 (0.0000995)	0.59 (0.1881505)	4.32 (6.60403E-08)	2.54 (0.0055082)
*BjNADH-GOGAT*	NADH dependent-glutamate synthase	0.01 (0.0015349)	0.78 (0.0009248)	0.57 (0.0031204)	0.13 (1.0008E-05)	0.08 (5.007965E-06)	0.16 (0.2714287)	0.33 (0.0015349)	1.24 (0.0009248)
*BjGDH1*	Glutamate dehydrogenase 1	1.22 (0.0050807)	17.71 (0.0009439)	2.02 (0.0494327)	2.26 (0.0064275)	0.86 (0.2454448)	0.9 (0.0012709)	2.72 (0.0050807)	0.7 (0.0009439)
*BjASN1*	Asparagine synthetase1	0.65 (0.0020390)	2.98 (1.53235E-06)	0.63 (0.0007311)	0.64 (0.0045533)	0.16 (6.29226E-08)	14.74 (0.0010291)	0.55 (0.0020390)	14.74 (0.0053235)
*BjASN2*	Asparagine synthetase 2	0.59 (0.0059720)	0.75 (0.0020124)	0.65 (3.63228E-06)	2.83 (0.0007120)	1.13 (0.2406302)	0.84 (0.0111437)	1.74 (0.0059720)	0.69 (0.0020124)
*BjPK*	Pyruvate kinase	0.78 (0.0062172)	0.78 (0.0128922)	0.65 (0.0111387)	0.23 (3.86170E-05)	4.47 (0.0003790)	0.78 (0.0109512)	7.05 (0.0062172)	3.36 (0.0128922)
*BjPePc*	Phosphoenolpyruvate carboxylase	0.99 (0.0086365)	0.93 (0.0444658)	0.72 (0.0649146)	0.89 (0.3959099)	1.35 (0.0779458)	1.03 (0.6548932)	1.57 (0.0086365)	1.17 (0.0444658)

### Effect of nitrogen depletion on expression of genes

To further check the importance of nitrogen source in regulating gene expression, we analyzed the expression of genes when seedlings were grown on medium devoid of nitrogen source (+Suc-N) (Fig 3B, S2C and S2D Fig). For this, we have compared expression of genes in +Suc-N condition w.r.t +Suc+N condition. A total of nine genes in root tissue and eleven genes in shoot tissue were significantly (P<0.05) downregulated when nitrogen was absent in the medium (Fig 4B, Table 1). In root tissue, expression of *BjNRT1*.*1*, *BjNRT1*.*5*, *BjNRT1*.*8*, *BjNRT2*.*1*, *BjAMT1*.*2*, *BjAMT2*, *BjNR2*, *BjGS1*.*1*, *BjGS2* was found to be downregulated, while expression of *BjNRT1*.*2*, *BjNRT1*.*3*, *BjNRT1*.*7*, *BjAMT1*.*1* and *BjGDH1* was found to be upregulated (Fig 3B, S2C and S2D Fig, Table 1). However, in case of shoot tissue, expression of most of the genes involved in nitrate reduction (*BjNR1*, *BjNR2*) and ammonium assimilation (*BjGS1*.*1*, *BjGS1*.*2*, *BjFd-GOGAT*, *BjNADH-GOGAT*) were found to be significantly downregulated (P<0.5) (Fig 3B, S2D Fig, Table 1). This showed that majority of N-assimilatory genes in shoot tissue were downregulated when grown in the media devoid of N source. The expression of *BjPK* in shoot tissue was found to be significantly downregulated under nitrogen depletion, however, the expression of *BjPePC* gene was found to be unchanged (Fig 3B, S2D Fig, Table 1).

### Effect of sucrose alone on expression of genes

**Fig 5:**
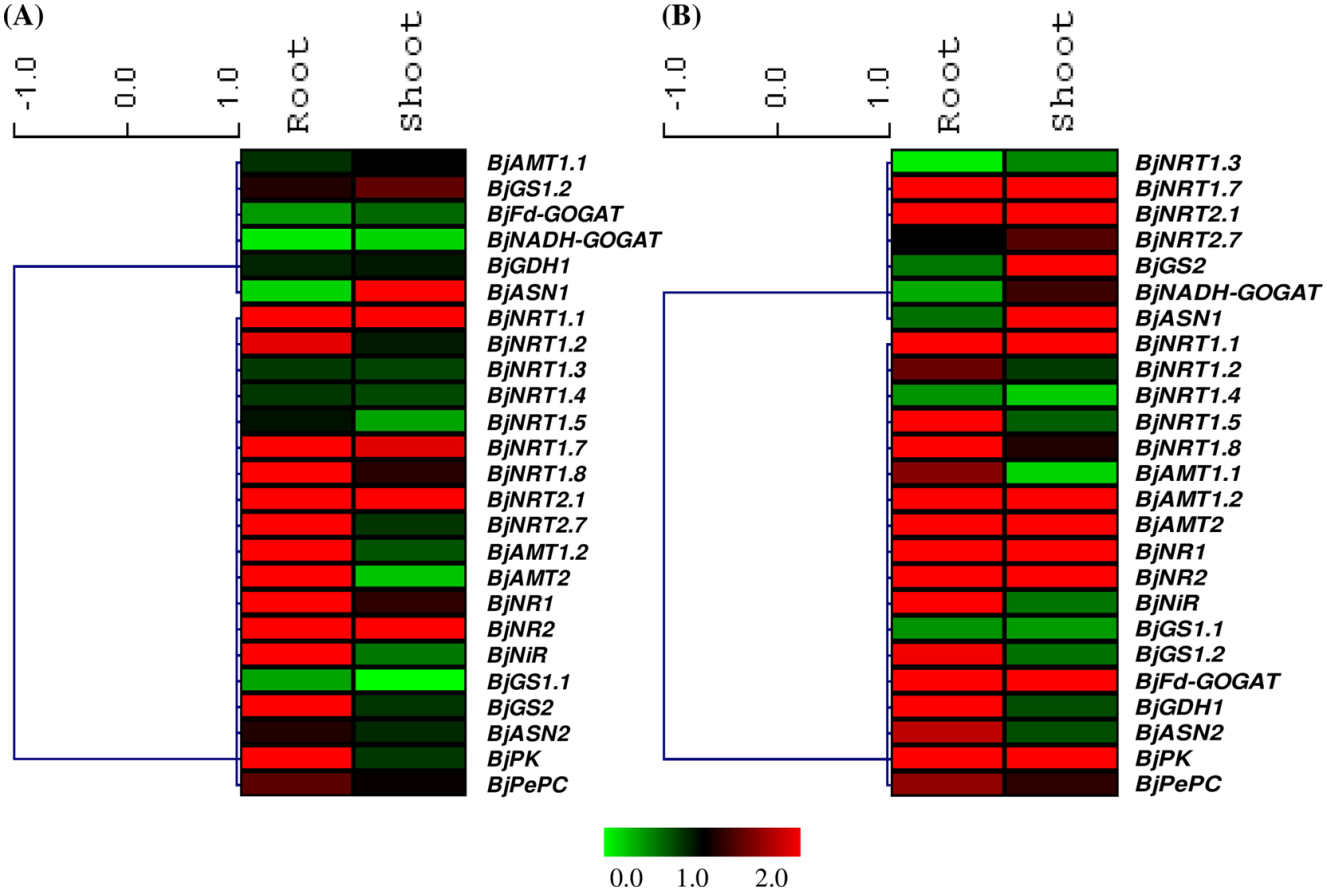
Heat maps showing relative expression of genes involved in nitrogen uptake, assimilation and gene of organic acid metabolism in root and shoot tissues of *B*. *juncea* in presence of carbon source alone (+Suc-N) w.r.t -Suc-N condition (A) and in presence of both carbon and sucrose source (+Suc+N) w.r.t -Suc-N condition (B). The vertical bars indicate relative expression ratio where red, black and green represent upregulation, no change and downregulation in transcript levels, respectively.

**Fig 6:**
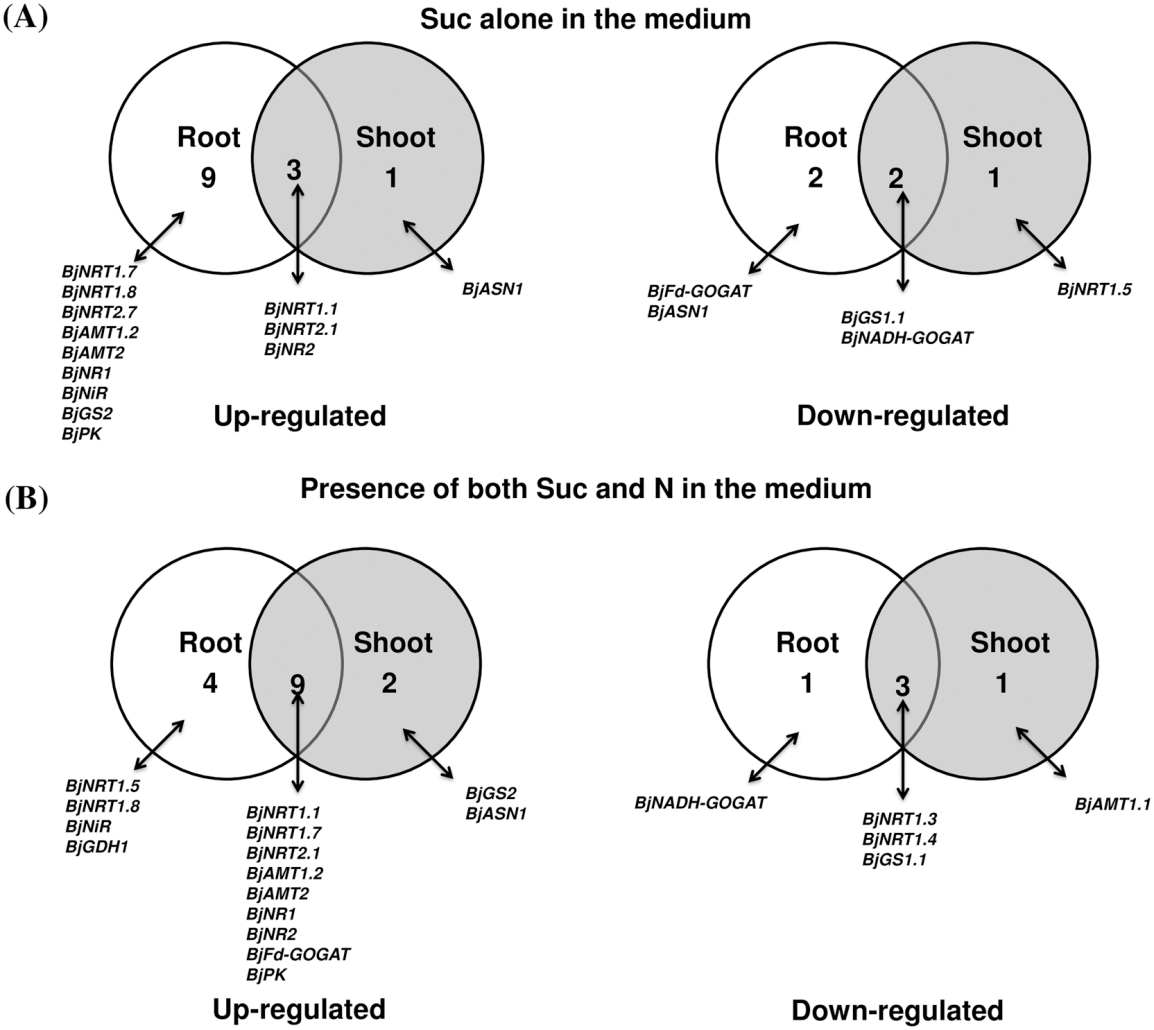
Venn diagram showing number of genes up-regulated or downregulated in root and shoot tissues in presence of Sucrose (Suc) alone in the medium (A) and in presence of both Nitrogen (N) and (Suc) in the medium (B). Genes with a relative fold change ≥2 and ≤0.5 with respect to control conditions were considered as up-regulated and down-regulated, respectively.

To study the effect of sucrose (carbon source) alone on the expression of genes involved in N-metabolism and organic acid metabolism, we compared the expression of genes in +Suc-N condition with -Suc-N condition. The expression pattern revealed that the expression of majority of genes of N-pathway was modulated under exogenous supply of sucrose (Fig 5A, S2E and S2F Fig, Table 1). The expression of sixteen genes in root (twelve induced and four repressed) and seven genes in shoot (four induced and three repressed) was significantly (P<0.05) modulated by sucrose alone (Fig 6A, Table 1). In root tissue, expression of several genes encoding for NRT (*BjNRT1*.*1*, *BjNRT1*.*7*, *BjNRT1*.*8*, *BjNRT2*.*1*, *BjNRT2*.*7*) and AMT transporters (*BjAMT1*.*2*, *BjAMT2*), nitrate reductase and nitrite reductase (*BjNR1*, *BjNR2*, *BjNiR*), cytosolic GS (*BjGS2*) and pyruvate kinase (*BjPK*) was significantly (p<0.05) upregulated and expression of *BjGS1*.*1*, *BjFd-GOGAT*, *BjNADH-GOGAT* was significantly (p<0.05) downregulated in presence of sucrose alone (Fig 5A, S2E and S2F Fig, Table 1). However, in shoot tissue, expression of four genes (*BjNRT1*.*1*, *BjNRT2*.*1*, *BjNR2* and *BjASN1*) was significantly upregulated and four were significantly downregulated (*BjNRT1*.*5*, *BjAMT2*, *BjGS1*.*1* and *BjNADH-GOGAT*) by sucrose alone (Fig 5A, Table 1). The expression of three genes (*BjAMT1*.*2*, *BjAMT2* and *BjPK*) in root and two genes (*BjAMT2*, *BjGS1*.*1*) in shoot was specifically regulated only by presence of sucrose, whereas these genes showed no change in expression in presence of exogenously supplied nitrogen.

### Regulation of gene expression by interaction of nitrogen and sucrose

Nitrogen and carbon interact with each other in order to regulate expression of various genes. We sought to identify those genes that are regulated by interaction of both, nitrogen and sucrose in the medium. For this, we have compared the condition +Suc+N with -Suc-N. The expression analysis showed that several genes of N-transport and assimilation pathways and genes involved in organic acid metabolism were regulated by interaction of both nitrogen and sucrose in the medium (Fig 5B, S2G and S2H Fig, Table 1). Expression of seventeen genes in root (thirteen induced and four repressed) and fifteen genes in shoot (eleven induced and four repressed) were found to be modulated when both nitrogen and sucrose were supplied in the medium (Fig 6B, Table 1). Expression of several genes, like *BjGS1*.*2* and *BjGDH1* in root and *BjGS2*, *BjFd-GOGAT* and *BjPK* in shoot was found to be significantly (P<0.05) upregulated only when both, sucrose and nitrogen source were present in the medium (Fig 5B, S2H Fig, Table 1). We have compared the expression pattern of all the genes in both root and shoot tissue in presence of N alone, Suc alone and when both are present in the medium and found that the expression of genes like, *BjNRT1*.*1*, *BjNRT2*.*1*, *BjNR2*, *BjNiR* in both root and shoot tissues were found to be modulated by sucrose and nitrogen alone and also when both are present together. In root tissue, the regulatory effect of nitrogen on expression of *BjNRT1*.*4*, *BjAMT1*.*2*, *BjAMT2*, *BjFD-GOGAT*, *BjGDH1* and *BjPK* was found to be sucrose dependent. On the other hand, the regulatory effect of sucrose on many genes of nitrogen metabolism (*BjNRT1*.*3*, *BjNRT1*.*4*, *BjNRT1*.*7*, *BjAMT1*.*2*, *BjNR1*, *BjNIR*, *BjGS1*, *BjGS2*, *BjFd-GOGAT* and *BjPK*) in shoot was found to be nitrogen dependent.

## Discussion

### Growth of *B*. *juncea* seedlings is affected by carbon and nitrogen availabilities

Carbon and nitrogen are essential for plant’s overall development [7]. In the present study, the effect of C/N availabilities on the growth and development of *B*. *juncea* seedlings was analyzed. We found that growth of *B*. *juncea* seedlings was affected by both the nutrients. Nitrogen can exert significant effect on root and shoot growth, as its deficiency increase root surface area and decrease shoot growth, on the other hand nitrogen supply supports shoot growth [39–41]. In the present study, we have observed that nitrogen alone can exert a significant effect in determining cotyledon size and growth of *B*. *juncea* seedlings. The increase in anthocyanin content in *B*. *juncea* seedling under absence of both, carbon and nitrogen may be due to nutrient deficiency in plants, as anthocyanin is secondary metabolite and its accumulation was found to be induced under nutrient deficiency [42].

The carbon and nitrogen pathways are highly interconnected, where reductants and energy produced from carbohydrate metabolism are used by N-assimilatory enzymes to perform N assimilation in plants [5]. This interaction between carbon and nitrogen pathways has prompted us to investigate the effect of exogenous sucrose and nitrogen on the expression of genes involved in N-uptake and assimilation and also the regulation of gene expression by interaction of these two nutrients. For inorganic nitrogen source, we have provided nitrate and ammonium and for carbon source, sucrose was added in the medium. Our results showed that nitrogen and sucrose regulate expression of genes involved in N-uptake and assimilation. Moreover, our results showed that expression of several genes of N-pathway is also regulated by exogenous sucrose supply. Previously studies on *Arabidopsis* seedlings in response to carbon and nitrogen treatments has revealed the presence of CN responsive *cis*-elements named as a nitrogen dependent enhancers of carbon regulation (NDEs) in the promoter region of the genes that respond to carbon and nitrogen [24]. This present study has suggested that differential behaviour of several genes of N-uptake and assimilatory pathway in response to nitrogen or sucrose alone or in combination may be due to presence of different CN-responsive elements in the promoter regions of these genes.

### Nitrogen and sucrose regulate genes of N-pathway in an organ dependent manner

The majority of genes in plant system respond to a particular environmental condition in an organ-dependent manner [43–44]. In our data, a distinct expression pattern of genes was observed in both root and shoot tissues in response to exogenously supplied nitrogen and/or carbon source. We have observed that exogenously supplied nitrogen alone can exert both, repressive and inductive effects in root tissue, whereas in case of shoot tissue only inductive effect of nitrogen was observed. In most of the plants, it has been observed that ammonium assimilation takes place directly in roots [45] and nitrate reduction takes place mainly in shoot tissue. However, a significant nitrate reduction/assimilation has been observed in nitrogen deprived *Arabidopsis* roots when transferred to nitrogen rich medium [46]. In present study, a strong upregulation in the expression of nitrate reductase and nitrite reductase genes in root tissue was observed which might suggest that nitrate reduction also take place in roots of *B*. *juncea*. It is also well known that genes of N-metabolism are negatively regulated by downstream products of N-assimilation [47,48]. Therefore, repression of some of the genes in root tissue in presence of N may be due to some downstream metabolites produced from ammonium assimilation process. Several studies in *Arabidopsis* have shown increased root growth in presence of exogenously supplied sucrose [49,50]. We have observed that exogenous supply of sucrose leads to induction of majority of N-uptake and assimilatory genes in roots of *B*. *juncea* as compared to shoot tissue. Exogenous supply of sucrose was found to increase the plant root growth in *Arabidopsis* [49,50]. Moreover, absence of nitrogen source in the media also leads to change in root system architecture mainly by remobilising nitrogen from shoot to root [38]. Sucrose is the major photosynthetic product and it provides energy as well as carbon skeleton for amino acid biosynthesis. Moreover, several enzymes of nitrogen assimilation were also found to be induced in presence of sucrose [22,23,51]. Taken together, these observations indicate that the induction of genes of N-uptake and assimilation pathway in root tissue may help *B*. *juncea* in optimising root growth when sucrose alone is present in the media.

### Transcriptional response of N-uptake and assimilatory genes and genes of organic acid pathway under nitrogen treatment

Nitrate and ammonium are two important nitrogen sources [52,53] and both can act as signal in order to regulate gene expression [46]. The present study has shown the importance of inorganic nitrogen source in regulating expression of genes involved in its uptake and assimilation. As nitrate is the main nitrogen source in most of the agricultural soils, a lot of microarray studies have been performed to understand the regulation of gene expression by nitrate in *A*. *thaliana* [13,14,16,54]. The ammonium has also been shown to influence gene expression in barley and rice [55,56]. In our data, the gene encoding dual-affinity nitrate transporter (NRT1.1) and enzyme nitrite reductase (NiR) showed strong upregulation by exogenously supplied nitrogen in root tissue. Whereas, in shoot tissue, *BjNRT2*.*1* and *BjGDH1* were found to be highly upregulated. Previous studies have shown upregulation in the expression of genes like nitrate transporter (*NRT2*.*1*), nitrate and nitrite reductase (*NR1*, *NR2*, *NiR*), glutamate synthase (*NADH-GOGAT*) and asparagine synthetase (*ASN2*) by nitrate supply [13,14,18]. However, we have identified several other genes of N-uptake and assimilation that were strongly upregulated by exogenous nitrogen supply. The expression of some of the genes, like *BjNRT1*.*7*, *BjNRT1*.*8*, *BjAMT1*.*1*, *BjAMT1*.*2*, *BjNR1* and *BjGDH1* in shoot tissue was found to be induced in presence of nitrogen alone, while no change in expression was observed when sucrose alone was present. This reflects that regulation of these genes solely depends on inorganic nitrogen source. The importance of inorganic nitrogen in regulating N-uptake and assimilation process in *B*. *juncea* can also be observed by the fact that absence of inorganic nitrogen in the media leads to downregulation of the expression of key N-assimilatory genes in *B*. *juncea*.

### Transcriptional response of N-uptake and assimilatory genes and genes of organic acid pathway under sucrose treatment

In plants, both sugars and inorganic nitrate are involved in adjusting nitrogen and carbon balance [57–59]. Sucrose is the most important carbohydrate synthesised and transported in the phloem of many plants [60,61]. Sucrose can act as signalling molecule in plants and it was found to regulate expression of several genes [62,63]. Coruzzi and Zhou [57] showed that carbon and nitrogen have matrix effect in plants which means that reduced carbon source leads to upregulation or downregulation of the genes of N- assimilation pathway, when present in abundance or scarce. Global transcriptional profiling of *Arabidopsis* has revealed that exogenous glucose regulates genes of nitrogen assimilation and amino acid metabolism more profoundly than nitrogen [18]. Also the mRNA levels of NR, GS1, GS2 and ASN1 were found to be affected by sucrose treatment in *Arabidopsis* [22,23,51]. The present study has also revealed that sucrose alone can affect expression of several genes of N-uptake and assimilation pathway in both, root and shoot tissues in *B*. *juncea*. In plants, the uptake of nitrate is not only regulated by N status, but C metabolites produced from photosynthesis also regulate this process [19,64,65]. In present study, the expression of several genes involved in nitrate uptake (*NRT1*.*1*, *NRT2*.*1*) and its translocation (*NRT1*.*7*, *NRT1*.*8*) from root to shoot was found to be upregulated in presence of sucrose alone. Several studies in plants, like *Arabidopsis*, barley, citrus and maize have also shown induction of nitrate and ammonia transporters in presence of sucrose [66–68]. In *Arabidopsis*, the induction of genes involved in N-transport by sucrose is linked with oxidative pentose phosphate metabolism (OPPP) and *cis*-acting elements in the promoter region of some genes [69,70]. The induction of genes encoding for nitrate reductase (*NR2* in both, root and shoot) and for nitrite reductase (*NiR* in root) in presence of sucrose alone in *B*. *juncea* was also observed, which may be due to the fact that the enzymes involved in nitrate reduction requires NADH and reduced ferredoxin that come from carbohydrate metabolism, addition of sucrose may in turn increase the supply of these reductants that may lead to induction of these genes.

### Several genes of N-assimilation pathway and genes of organic acid metabolism are regulated by interaction of nitrogen and carbon

Microarray analysis of *A*. *thaliana* in response to carbon and nitrogen has revealed that plant metabolic processes were highly influenced by the interaction of carbon and nitrogen signalling pathways [24]. In addition, several studies on *Arabidopsis* and rice have revealed that both carbon and nitrogen interact with each other for regulating gene expression [25,70,71]. The expression of *ASN1* and *GLN2* in *Arabidopsis* was found to be reciprocally regulated by carbon (or light) and nitrogen [22,23]. The importance of carbon and nitrogen interaction in regulating gene expression in *B*. *juncea* can be observed by the fact that majority of N-uptake and assimilatory genes were found to respond when both of these nutrients were present in the media. The *AtNRT1*.*1* is the first regulatory gene involved in nitrate signalling and encodes a dual affinity nitrate transporter [72,73]. The AtNRT1.1 transporter also acts as nitrate sensor and can regulate several downstream nitrate regulatory signalling mechanisms [74]. In addition, AtNRT2.1 was also shown to act as a nitrate sensor [75]. In the present analysis, both *NRT1*.*1* and *NRT2*.*1* were found to be induced in response to nitrogen or sucrose alone and also in presence of both, which may suggest their involvement in crosstalk between carbon and nitrogen signalling mechanisms in *B*. *juncea*. In *A*. *thaliana*, a *cis*-acting element in *AtNRT2*.*1* promoter was found to be involved in response to exogenous NO_3_^-^ and sugar [70]. Another gene that was found to be induced in both, root and shoot tissues by nitrogen and sucrose alone and together was *BjNR2* that encodes for nitrate reductase. Previously, expression of gene encoding nitrate reductase was found to be regulated by light, nitrate and sugars [59,76]. The upregulation of *BjNR2* by nitrogen and sucrose alone and together in *B*. *juncea* might also reflect its involvement in both carbon and nitrogen metabolic pathways. In plants, both glutamine synthetase (GS) and glutamate synthase (GOGAT) play major role in ammonium assimilation, whereas pyruvate kinase is the rate limiting enzyme in carbon metabolism. These enzymes are known to be involved in tight regulation of carbon and nitrogen metabolisms [3,77]. Up-regulation in the expression of *BjGS1*.*2*, *BjGDH1 (*Root), *BjGS2*, *BjFd-GOGAT* and *BjPK* genes (shoot) in *B*. *juncea*, when both sucrose and nitrogen are present may reflect their importance in maintaining tight coordination of carbon and nitrogen metabolism in *B*. *juncea*.

## Conclusions

The present work was carried out to understand the role of carbon and nitrogen in regulating growth of *B*. *juncea* seedlings and also to study the regulation of gene expression by both the nutrients. The presence of N source alone in the medium was found to increase cotyledon size and shoot length of *B*. *juncea* seedlings. Role of nitrate in regulating gene expression is well known, however our analysis revealed that sucrose alone can also regulate expression of genes of nitrogen metabolism that were not identified in previous studies. Both, carbon and nitrogen sources were found to regulate gene expression, either positively or negatively. Distinct expression patterns in both root and shoot tissues in response to nitrogen and sucrose was observed which showed that the regulation of gene expression by these nutrients is organ specific in *B*. *juncea*. In presence of sucrose, the expression of genes in root tissue was found to be affected more as compared to shoot tissue. Our study also suggests that both, nitrogen and sucrose can modulate the effect of each other in regulating gene expression. The expression data has revealed several common genes of N- metabolism in both root and shoot tissue that are affected when both, nitrogen and sucrose are present in the medium indicating importance of C/N signalling in regulating gene expression (S2 Table). In present work, differential expression pattern of various genes in response to carbon and nitrogen may be due presence of different *cis*-acting elements in their promoter regions. From our study, it is clear that as a signal, inorganic nitrogen or sucrose alone can regulate expression of genes involved in N-transport and assimilation, but how much of this inorganic nitrogen or sucrose change the downstream metabolites level is, not clear. Moreover, the key components involved in C/N sensing and signalling are not clearly known, due to complexity in the plant system. Therefore, understanding the exact molecular mechanism involved downstream to C/N signalling transduction pathway will be interesting, in future.

## Supporting Information

S1 Fig Seeds of *B*. *juncea* were germinated on modified MS medium in absence of both carbon and nitrogen source (-Suc-N) (A), in presence of sucrose alone (+Suc-N) (B), in presence of nitrogen source alone (-Suc+N) (C) and in presence of carbon and nitrogen source (+Suc+N) (D). Increased cotyledon size and shoot length of *B*. *juncea* seedlings grown on–Suc+N and +Suc+N media was observed as compared to seedlings grown on–Suc-N and +Suc-N media (E). For shoot length, comparison seedlings grown on different medium were placed in single vertical petriplates and photographs were taken.
(PPTX)

S2 Fig Bar diagrams showing relative expression of genes involved in N-uptake and assimilation and genes of organic acid metabolism in root and shoot tissue of *B*. *juncea* in presence of inorganic nitrogen alone (-Suc-N vs.-Suc+N) (A, B), in absence of inorganic nitrogen (+Suc+N vs.+Suc-N) (C, D), in presence of sucrose alone (-Suc-N vs.+Suc-N) (E, F) and in presence of both inorganic nitrogen and sucrose (-Suc-N vs. +Suc+N) (G, H). Asterisks on the top of the bar indicate statistically significant differences (*P-value <0.05).
(PPTX)

S1 Table List of primers used for qRT-PCR analysis and their amplicon size. (XLSX)

S2 Table List of genes commonly affected (up and down-regulated) in both root and shoot tissue of *B*. *juncea* L. under different C and N availabilities i.e. N alone in the medium, N depletion in the medium, Suc alone in the medium and presence of both N and Suc in the medium. (DOCX)

## References

[1] Kusano M, Fukushima A, Redestig H, Saito K. Metabolomic approaches toward understanding nitrogen metabolism in plants. J Exp Bot. 2011;62: 1439–1453. 10.1093/jxb/erq417 21220784

[2] Stitt M, Krapp A. The molecular physiological basis for the interaction between elevated carbon dioxide and nutrients. Plant Cell Environ. 1999;22: 583**–**622.

[3] Zheng ZL. Carbon and nitrogen nutrient balance signalling in plant. Plant Signal Behav. 2009;4: 584–591. 19820356 10.4161/psb.4.7.8540 PMC2710548

[4] Coruzzi G, Bush DR. Nitrogen and carbon nutrient and metabolite signaling in plants. Plant Physiol. 2001;125: 61–64. 11154297 10.1104/pp.125.1.61 PMC1539326

[5] Nunes-nesi N, Fernie AR, Stitt M. Metabolic and signalling aspects underpinning the regulation of plant carbon nitrogen interaction. Mol Plant. 2010;3: 973–996. 10.1093/mp/ssq049 20926550

[6] Signora L, De Smet I, Foyer CH, Zhang H. ABA plays a central role in mediating the regulatory effects of nitrate on root branching in *Arabidopsis*. Plant J. 2001;28: 655–662. 11851911 10.1046/j.1365-313x.2001.01185.x

[7] Martin T, Oswald O, Graham IA. *Arabidopsis* seedling growth, storage lipid mobilization and photosynthetic gene expression are regulated by carbon: nitrogen availability. Plant Physiol. 2002;128: 472–481. 11842151 10.1104/pp.010475 PMC148910

[8] Miller AJ, Crammer MD. Root nitrogen acquisition and assimilation. Plant Soil. 2004;274: 1–36.

[9] Jorgensen I, Stiles W. Carbon assimilation. New Phytol. 1916;15: 176–193.

[10] Ourry A, Macduff JH, Volenec JJ, Gaudillère JP. Nitrogen traffic during plant growth and development In: Morot-Gaudry JF, Lea P, editors. Plant nitrogen. Berlin: Springer Verlag; 2001 pp. 255–273.

[11] Foyer CH, Noctor G, Hodges M. Respiration and nitrogen assimilation: targeting mitochondria-associated metabolism as a means to enhance nitrogen use efficiency. J Exp Bot. 2011;62: 1467–1482. 10.1093/jxb/erq453 21282329

[12] Raab TK, Terry N. Nitrogen source regulation of growth and photosynthesis in *Beta vulgaris* L. Plant Physiol. 1994;105: 1159–1166. 12232273 10.1104/pp.105.4.1159 PMC159444

[13] Wang R, Guegler K, LaBrie ST, Crawford NM. Genomic analysis of a nutrient response in *Arabidopsis* reveals diverse expression patterns and novel metabolic and potential regulatory genes that are induced by nitrate. Plant Cell. 2000;12: 1491–1510. 10948265 10.1105/tpc.12.8.1491 PMC149118

[14] Wang R, Okamoto M, Xing X, Crawford NM. Microarray analysis of the nitrate response in *Arabidopsis* roots and shoots reveals over 1,000 rapidly responding genes and new linkages to Glucose, Trehalose-6-Phosphate, Iron, and Sulfate Metabolism. Plant Physiol. 2003;132: 556–567. 12805587 10.1104/pp.103.021253 PMC166997

[15] Wang R, Tischner R, Gutierrez RA, Hoffman M, Xing X, Chen M, et al Genomic analysis of the nitrate response using a nitrate reductase-null mutant of *Arabidopsis*. Plant Physiol. 2004;136: 2512–2522. 15333754 10.1104/pp.104.044610 PMC523318

[16] Scheible WR, Morcuende R, Czechowski T, Fritz C, Osuna D, Palacios-Rojas N, et al Genome-wide reprogramming of primary and secondary metabolism, protein synthesis, cellular growth processes, and the regulatory infrastructure of *Arabidopsis* in response to nitrogen. Plant Physiol. 2004; 136: 2483–2499. 15375205 10.1104/pp.104.047019 PMC523316

[17] Gibon Y, Blasing OE, Palacios N, Pankovic D, Hendriks JHM, Fisahn J, et al Adjustment of diurnal starch turnover to short days: Depletion of sugar during the night leads to a temporary inhibition of carbohydrate utilization, accumulation of sugars and post-translational activation of ADP-glucose pyrophosphorylase in the following light period. Plant J. 2004;39: 847–862. 15341628 10.1111/j.1365-313X.2004.02173.x

[18] Price J, Laxmi A, St Martin SK, Jang JC. Global transcription profiling reveals multiple sugar signal transduction mechanisms in *Arabidopsis*. Plant Cell. 2004;16: 2128–2150. 15273295 10.1105/tpc.104.022616 PMC519203

[19] Lejay L, Gansel X, Cerezo M, Tillard P, Muller C, Krapp A, et al Regulation of root ion transporters by photosynthesis: functional importance and relation with hexokinase. Plant Cell. 2003;15: 2218–2232. 12953122 10.1105/tpc.013516 PMC181342

[20] Li Y, Lee K, Walsh S, Smith C, Hadingham S, Sorefan K, et al Establishing glucose- and ABA-regulated transcription networks in *Arabidopsis* by microarray analysis and promoter classification using a Relevance Vector Machine. Genome Research. 2006;16; 414–427. 16424108 10.1101/gr.4237406 PMC1415219

[21] Matt P, Krapp A, Haake V, Mock HP, Stitt M. Decreased Rubisco activity leads to dramatic changes of nitrate metabolism, amino acid metabolism and the levels of phenylpropanoids and nicotine in tobacco antisense RBCS transformants. Plant J. 2002;30: 663–677. 12061898 10.1046/j.1365-313x.2002.01323.x

[22] Lam HM, Peng SS, Coruzzi GM. Metabolic regulation of the gene encoding glutamine-dependent asparagine synthetase in *Arabidopsis thaliana*. Plant Physiol. 1994;106: 1347–1357. 7846154 10.1104/pp.106.4.1347 PMC159672

[23] Oliveira IC, Coruzzi GM. Carbon and amino acids reciprocally modulate the expression of glutamine synthetase in *Arabidopsis*. Plant Physiol. 1999;121: 301–309. 10482686 10.1104/pp.121.1.301 PMC59385

[24] Palenchar PM, Kouranov A, Lejay LV, Coruzzi GM. Genome-wide patterns of carbon and nitrogen regulation of gene expression validate the combined carbon and nitrogen (CN)-signaling hypothesis in plants. Genome Biol. 2004;5: R91 15535867 10.1186/gb-2004-5-11-r91 PMC545782

[25] Gutierrez RA, Lejay LV, Dean A, Chiaromonte F, Shasha DE, Coruzzi GM. Qualitative network models and genome-wide expression data define carbon/nitrogen-responsive molecular machines in *Arabidopsis*. Genome Biol. 2007;8: R7 17217541 10.1186/gb-2007-8-1-r7 PMC1839130

[26] Malamy JE, Ryan KS. Environmental regulation of lateral root initiation in *Arabidopsis*. Plant Physiol. 2001;127: 899–909. 11706172 PMC129261

[27] Kang J, Turano FJ. The putative glutamate receptor 1.1 (AtGLR1.1) functions as a regulator of carbon and nitrogen metabolism in *Arabidopsis thaliana*. Proc Natl Acad Sci U S A. 2003;100: 6872–6877. 12738881 10.1073/pnas.1030961100 PMC164539

[28] Gao P, Xin Z, Zheng ZL. The OSU1/QUA2/TSD2-encoded putative methyltransferase is a critical modulator of carbon and nitrogen nutrient balance response in *Arabidopsis*. PLoS ONE. 2008;3: e1387 10.1371/journal.pone.0001387 18167546 PMC2148111

[29] Uprety DC, Rabha BK. Effects of elevated CO_2_ and moisture stress on *Brassica juncea*. Photosynthetica. 1998;35: 597–602.

[30] Uprety DC, Mahalaxmi V. Effect of elevated CO_2_ and nitrogen nutrition on photosynthesis, growth and carbon nitrogen balance in *Brassica juncea*. J Agron Crop Sci. 2000;184: 271–276.

[31] Seth CS, Mishra V. Changes in C-N metabolism under elevated CO_2_ and temperature in Indian mustard (*Brassica juncea* L.): an adaptation strategy under climate change scenario. J Plant Res. 2014;127: 793–802. 10.1007/s10265-014-0664-9 25246072

[32] Murashige T, Skoog F. A revised medium for rapid growth and bioassays with tobacco tissue cultures. Physiol Plant. 1962;15: 473–497.

[33] Arnon DI. Copper enzymes in chloroplasts. Phenol oxidase in *Beta vulgaris*. Plant Physiol. 1949;24: 1–15. 16654194 10.1104/pp.24.1.1 PMC437905

[34] Mancinelli AL, Hoff AM, Cottrell M. Anthocyanin production in Chl-rich and Chl-poor seedlings. Plant Physiol. 1988;86: 652–654. 16665964 10.1104/pp.86.3.652 PMC1054546

[35] Ghawana S, Paul A, Kumar H, Kumar A, Singh H, Bhardwaj PK, et al An RNA isolation system for plant tissues rich in secondary metabolites. BMC Res Notes. 2011;4: 85 10.1186/1756-0500-4-85 21443767 PMC3079660

[36] Goel P, Singh AK. Abiotic stresses downregulate key genes involved in nitrogen uptake and assimilation in *Brassica juncea* L. PLoS One. 2015;10: e0143645 10.1371/journal.pone.0143645 26605918 PMC4659633

[37] Chandna R, Augustine R, Bisht NC. Evaluation of candidate reference genes for gene expression normalization in *Brassica juncea* using real time quantitative RT-PCR. PLoS One. 2012;5: e36918. 10.1371/journal.pone.0036918 PMC3350508 22606308

[38] Livak K. J. & Schmittgen T. D. Analysis of relative gene expression data using real-time quantitative PCR and the 2(-Delta Delta C(T)) Method. Methods. 2001; 25: 402–408. 11846609 10.1006/meth.2001.1262

[39] Scheible WR, Lauerer M, Schulze ED, Caboche M, Stitt M. Accumulation of nitrate in the shoot acts as a signal to regulate shoot-root allocation in tobacco. Plant J. 1997;11: 671–691.

[40] Lloert F, Casanovas C, Penuelas J. 1999 Seedling survival of mediterranean shrub land species in relation to root: shoot ratio, seed size and water and nitrogen use. Functional Ecol.1999;13: 210–216.

[41] Shangguan ZP, Shao MA, Ren SJ, Zhang LM, Xue Q. Effect of nitrogen on root and shoot relations and gas exchange in winter wheat. Bot Bull Acad Sin. 2004;45: 49–54.

[42] Steyn WJ, Wand SJE, Holcroft DM, Jacobs G. Anthocyanins in vegetative tissues: proposed unified function in photoprotection. New Phytol. 2002;155: 349–36. 10.1046/j.1469-8137.2002.00482.x 33873306

[43] Aceituno FF, Moseyko N, Rhee SY, Gutierrez RA. The rules of gene expression in plants: organ identity and gene body methylation are key factors for regulation of gene expression in *Arabidopsis thaliana*. BMC Genomics. 2008;9: 438 10.1186/1471-2164-9-438 18811951 PMC2566314

[44] Krapp A, Berthome R, Orsel M, Mercey-Boutet S, Yu A, Castaings L, et al *Arabidopsis* roots and shoots show distinct temporal adaptation patterns toward nitrogen starvation. Plant Physiol. 2011;157: 1255–1282. 10.1104/pp.111.179838 21900481 PMC3252138

[45] Bloom AJ, Meyerhoff PA, Taylor AR, Rost TL. Root development and absorption of ammonium and nitrate from the rhizosphere. Plant Growth Regul. 2003;21: 416–431.

[46] Patterson K, Cakmak T, Cooper A, Lager I, Rasmusson AG, Escobar MA. Distinct signalling pathways and transcriptome response signatures differentiate ammonium- and nitrate-supplied plant. Plant Cell Environ. 2010;9: 1486–1501. 10.1111/j.1365-3040.2010.02158.x PMC2920365 20444219

[47] Rawat SR, Silim SN, Kronzucker HJ, Siddiqi MY, Glass ADM. AtAMT1 gene expression and NH4+ uptake in roots of *Arabidopsis thaliana*: evidence for regulation by root glutamine levels. Plant J. 1999;19: 143–152. 10476061 10.1046/j.1365-313x.1999.00505.x

[48] Miller AJ, Fan X, Shen Q, Smith SJ. Amino acids and nitrate as signals for the regulation of nitrogen acquisition. J Exp Bot. 2007;59: 111–119. 18093964 10.1093/jxb/erm208

[49] Freixes S, Thibaud M, Tardieu F, Muller B. Root elongation and branching is related to local hexose concentration in *Arabidopsis thaliana* seedlings. Plant Cell Environ. 2002;25: 1357–1366.

[50] Roycewicz P, Malamy JE. Dissecting the effect of nitrate, sucrose and osmotic potential on *Arabidopsis* root and shoot system growth in laboratory assay. Phil Trans R SocLond B: Biol Sci. 2012;367: 1489–1500. 10.1098/rstb.2011.0230 PMC3321681 22527391

[51] Cheng CL, Acedo GN, Cristinsin M, Conkling MA. Sucrose mimics the light induction of *Arabidopsis* nitrate reductase gene transcription. Proc Natl AcadSci U S A. 1992;89: 1861–1864. 10.1073/pnas.89.5.1861 PMC48553 1542684

[52] Raven JA, Smith FA. Nitrogen assimilation and transport in vascular land plants in relation to intracellular pH regulation. New Phytol. 1976;76: 415–431.

[53] Mokhele B, Zhan X, Yang G, Zhang X. Review: nitrogen assimilation in crop plants and its affecting factors. Can J Plant Sci. 2012;92: 399–405.

[54] Bi YM, Wang RL, Zhu T, Rothstein SJ. Global transcription profiling reveals differential responses to chronic nitrogen stress and putative nitrogen regulatory components in *Arabidopsis*. BMC Genomics. 2007;8: 281 17705847 10.1186/1471-2164-8-281 PMC1994689

[55] Lopes MS, Araus JL. Comparative genomics and physiological analysis of nutrient response to NH4+ and No3- in barley seedlings. Physiol Plant. 2008;134: 134–150. 10.1111/j.1399-3054.2008.01114.x 18544123

[56] Zhu GH, Zhuang CX, Wang YQ, Jiang LR, Peng XX. Differential expression of rice genes under different nitrogen forms and their relationship with sulphur metabolism. J Integr Plant Biol. 2006;48: 1177–1184.

[57] Coruzzi GM., Zhou L., 2001 Carbon and nitrogen sensing and signaling in plants: Emerging ‘matrix effects’. Curr Opin Plant Biol.2002;4: 247–253. 11312136 10.1016/s1369-5266(00)00168-0

[58] Forde BG. 2002 Local and long-range signalling pathways regulating plant responses to nitrate. Annu Rev Plant Biol.2002;53: 203–224. 12221973 10.1146/annurev.arplant.53.100301.135256

[59] Stitt M, Muller C, Matt P, Gibon Y, Carillo P, Morcuende R, et al Steps towards an integrated view of nitrogen metabolism. J Exp Bot. 2002;53: 959–970. 11912238 10.1093/jexbot/53.370.959

[60] Thompson M, Thorpe T. Metabolic and non-metabolic roles of carbohydrates In: Bonga JM, Durzan DJ, editors. Cell and Tissue Culture in Forestry. Dordrecht: Martinus Nijhoff Publishers; 1987 pp. 89–112.

[61] Ahmad T, Abbasi NA, Hafiz IA, Ali A. Comparison of sucrose and sorbitol as main carbon energy sources in micropropagation of peach rootstock gf-677. Pak J Bot 2007;4: 1269–1275.

[62] Wind J, Smeekens S, Hanson J. Sucrose: metabolite and signalling molecule. Phytochemistry. 2010;71: 1610–1614. 10.1016/j.phytochem.2010.07.007 20696445

[63] Tognetti JA, Pontis HG, Martinez-Noel GMA. Sucrose signaling in plants: a world yet to be explored. Plant Signal behav. 2013;3: e23316. 10.4161/psb.23316 PMC3676498 23333971

[64] Lejay L, Tillard P, Lepetit M, Olive F, Filleur S, Daniel-Vedele F, et al Molecular and functional regulation of two NO_3_- uptake systems by N- and C-status of *Arabidopsis* plants. Plant J. 1999;18: 509–519. 10417701 10.1046/j.1365-313x.1999.00480.x

[65] Zhou S, Gap X, Wang C, Yang G, Cram WJ, He G. Identification of sugar signals controlling the nitrate uptake by rice roots using a noninvasive technique. Z Naturforsch C. 2009;64: 697–703. 19957439 10.1515/znc-2009-9-1015

[66] Lejay L, Wirth J, Pervent M, Cross JM, Tillard P, Gojon A. Oxidative pentose phosphate pathway-dependent sugar sensing as a mechanism for regulation of root ion transporters by photosynthesis. Plant Physiol. 2008;146: 2036–2053. 10.1104/pp.107.114710 18305209 PMC2287369

[67] Camanes G, Cerezo M, Millo-Primo E, Gojon A, Garcia-Agustin P. Ammonium transport and *CiAMT1* expression are regulated by light and sucrose in Citrus plants. J Exp Bot. 2007;58: 2811–2825. 17615410 10.1093/jxb/erm135

[68] Sehtiya HL, Goyal SS. Comparative uptake of nitrate by intact seedlings of C-3 barley) and C-4 (corn) plants: effect of light and exogenously supplied sucrose. Plant Soil. 2000;227: 185–190.

[69] De Jong F, Thodey K, Lejay LV, Bevan MW. Glucose elevates NITRATE TRANSPORTER 2.1 protein levels and nitrate transport activity independently of its HEXOKINASE1-mediated stimulation of NITRATE TRANSPORTER 2.1 expression. Plant Physiol. 2014;168: 308–320. 10.1104/pp.113.230599 PMC3875810 24272701

[70] Girin T, Lejay L, Wirth J, Widiez T, Palenchar PM, Nazoa P, et al Identification of a 150 bp cis-acting element of the AtNRT2.1 promoter involved in the regulation of gene expression by the N and C status of the plant. Plant Cell Environ. 2007;30: 1366–1380. 17897408 10.1111/j.1365-3040.2007.01712.x

[71] Sun W, Huang A, Sang Y, Fu Y, Yang Z. Carbon-Nitrogen interaction modulates plant growth and expression of metabolic genes in rice. J Plant Growth Regul. 2013;32: 575–584.

[72] Tsay YF, Schroeder JI, Feldmann KA, Crawford NM. The herbicide sensitivity gene CHL1 of *Arabidopsis* encodes a nitrate-inducible nitrate transporter. Cell. 1993;72: 705–713. 8453665 10.1016/0092-8674(93)90399-b

[73] Liu KH, Huang CY, Tsay YF. CHL1 is a dual-affinity nitrate transporter of *Arabidopsis* involved in multiple phases of nitrate uptake. Plant Cell. 1999;11: 865–874. 10330471 10.1105/tpc.11.5.865 PMC144217

[74] Gojon A, Krouk G, Perrine-Walker F, Laugier E. Nitrate transceptors in plant. J Exp Bot. 2011;62: 2299–2308. 10.1093/jxb/erq419 21239382

[75] Little DY, Rao H, Olivia S, Daniel-Vedele F, Krapp A, Malamy JE. The putative high-affinity nitrate transporter NRT2.1 represses lateral root initiation in response to nutritional cues. Proc Natl Acad U S A. 2005;102: 13693–13698. 10.1073/pnas.0504219102 PMC1224627 16157886

[76] Campbell WH. Nitrate reductase structure, function and regulation: Bridging the gap between biochemistry and physiology. Annu Rev Plant Physiol. 1999;50: 277–303. 10.1146/annurev.arplant.50.1.277 15012211

[77] Miflin BJ, Habash DZ. The role of glutamine synthetase and glutamate dehydrogenase in nitrogen assimilation and possibilities for improvement in the nitrogen utilization of crops. J Exp Bot. 2002;53: 979–987. 11912240 10.1093/jexbot/53.370.979

