# Supplementary material for: Carbon: Nitrogen Interaction Regulates Expression of Genes Involved in N-Uptake and Assimilation in Brassica juncea L
Source: PLoS One. 2016 Sep 16;11(9):e0163061. doi: 10.1371/journal.pone.0163061 (PMC5026376; doi:10.1371/journal.pone.0163061)
Supplement: S1 Fig — Seeds of B. juncea were germinated on modified MS medium in absence of both carbon and nitrogen source (-Suc-N) (A), in presence of sucrose alone (+Suc-N) (B), in presence of nitrogen source alone (-Suc+N) (C) and in presence of carbon and nitrogen source (+Suc+N) (D). Increased cotyledon size and shoot length of B. juncea seedlings grown on–Suc+N and +Suc+N media was observed as compared to seedlings grown on–Suc-N and +Suc-N media (E). For shoot length, comparison seedlings grown on different medium were placed in single vertical petriplates and photographs were taken. (PPTX) [file pone.0163061.s001.pptx]

## Slide 1
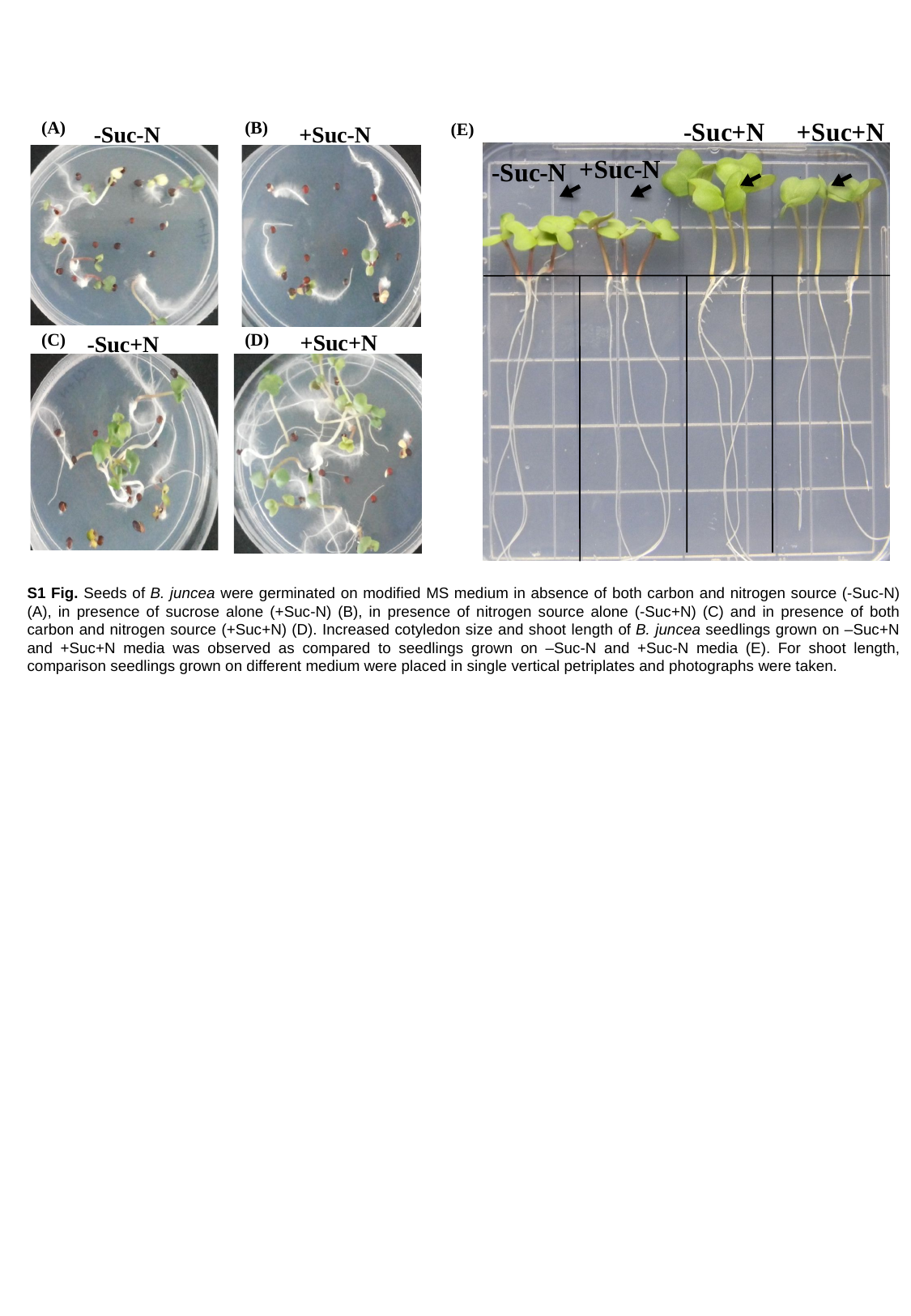

(B)
(A)
(E)
-Suc-N
+Suc-N
+Suc+N
(C)
(D)
-Suc+N
S1 Fig. Seeds of B. juncea were germinated on modified MS medium in absence of both carbon and nitrogen source (-Suc-N) (A), in presence of sucrose alone (+Suc-N) (B), in presence of nitrogen source alone (-Suc+N) (C) and in presence of both carbon and nitrogen source (+Suc+N) (D). Increased cotyledon size and shoot length of B. juncea seedlings grown on –Suc+N and +Suc+N media was observed as compared to seedlings grown on –Suc-N and +Suc-N media (E). For shoot length, comparison seedlings grown on different medium were placed in single vertical petriplates and photographs were taken.
