# Supplementary material for: Carbon: Nitrogen Interaction Regulates Expression of Genes Involved in N-Uptake and Assimilation in Brassica juncea L
Source: PLoS One. 2016 Sep 16;11(9):e0163061. doi: 10.1371/journal.pone.0163061 (PMC5026376; doi:10.1371/journal.pone.0163061)
Supplement: S2 Fig — Asterisks on the top of the bar indicate statistically significant differences (*P-value <0.05). (PPTX) [file pone.0163061.s002.pptx]

## Slide 1
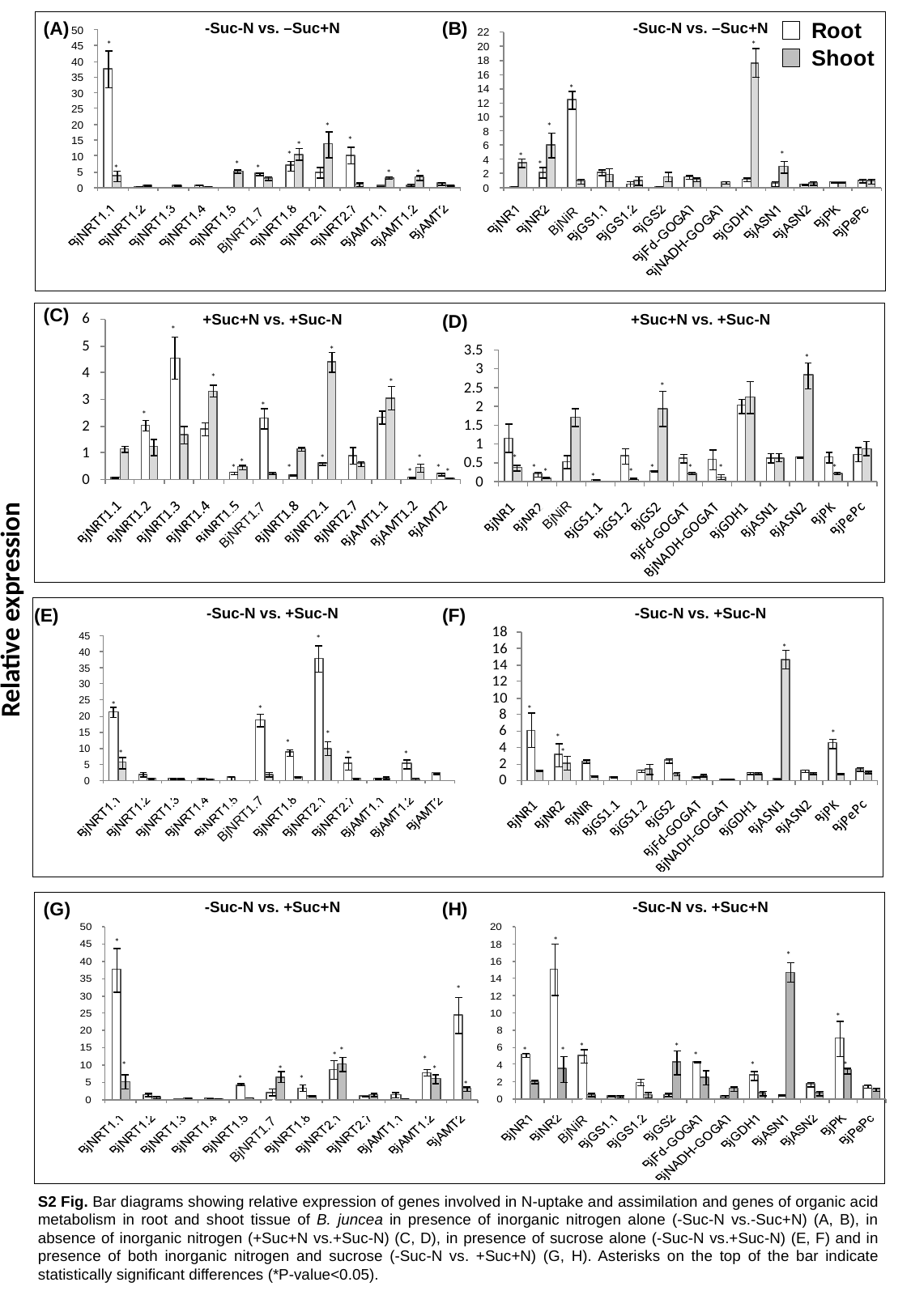

(A)
(B)
-Suc-N vs. –Suc+N
-Suc-N vs. –Suc+N
 *
 *
 *
 *
 *
 *
 *
 *
 *
 *
 *
 *
 *
 *
 *
 *
BjNiR
BjNRT1.7
(C)
(D)
+Suc+N vs. +Suc-N
+Suc+N vs. +Suc-N
 *
 *
 *
 *
 *
 *
 *
 *
 *
 *
 *
 *
 *
 *
 *
 *
 *
 *
 *
 *
 *
 *
 *
 *
 *
BjNiR
BjNRT1.7
Relative expression
(E)
(F)
-Suc-N vs. +Suc-N
-Suc-N vs. +Suc-N
 *
 *
 *
 *
 *
 *
 *
 *
 *
 *
 *
 *
 *
BjNRT1.7
(G)
(H)
-Suc-N vs. +Suc+N
-Suc-N vs. +Suc+N
 *
 *
 *
 *
 *
 *
 *
 *
 *
 *
 *
 *
 *
 *
 *
 *
 *
 *
 *
 *
 *
BjNiR
BjNRT1.7
S2 Fig. Bar diagrams showing relative expression of genes involved in N-uptake and assimilation and genes of organic acid metabolism in root and shoot tissue of B. juncea in presence of inorganic nitrogen alone (-Suc-N vs.-Suc+N) (A, B), in absence of inorganic nitrogen (+Suc+N vs.+Suc-N) (C, D), in presence of sucrose alone (-Suc-N vs.+Suc-N) (E, F) and in presence of both inorganic nitrogen and sucrose (-Suc-N vs. +Suc+N) (G, H). Asterisks on the top of the bar indicate statistically significant differences (*P-value<0.05).
