# Supplementary material for: Carbon: Nitrogen Interaction Regulates Expression of Genes Involved in N-Uptake and Assimilation in Brassica juncea L
Source: PLoS One. 2016 Sep 16;11(9):e0163061. doi: 10.1371/journal.pone.0163061 (PMC5026376; doi:10.1371/journal.pone.0163061)
Supplement: S2 Table — (DOCX) [file pone.0163061.s004.docx]

| **Category** | **N alone in the medium** | **N depletion in the medium** | **Suc alone in the medium** | **Presence of both Suc and N in the medium** |
| --- | --- | --- | --- | --- |
| Up-regulated | *BjNRT1.1*  *BjNRT1.7*  *BjNRT1.8*  *BjNRT2.1*  *BjNR2* | *BjAMT1.1*  *BjGDH1* | *BjNRT1.1*  *BjNRT2.1*  *BjNR2* | *BjNRT1.1*  *BjNRT1.7*  *BjNRT2.1*  *BjAMT1.2*  *BjAMT2*  *BjNR1*  *BjNR2*  *BjFd-GOGAT*  *BjPK* |
| Down-regulated |  | *BjNRT1.5*  *BjAMT1.2*  *BjAMT2*  *BjNR2*  *BjGS1.1* | *BjGS1.1*  *BjNADH-GOGAT* | *BjNRT1.3*  *BjNRT1.4*  *BjGS1.1* |

**S2 Table.** List of genes commonly affected (up and down-regulated) in both root and shoot tissue of *B. juncea* L. under different C and N availabilities i.e. N alone in the medium, N depletion in the medium, Suc alone in the medium and presence of both N and Suc in the medium.
